# Developmental Changes in Emotion Recognition from Full-Light and Point-Light Displays of Body Movement

**DOI:** 10.1371/journal.pone.0044815

**Published:** 2012-09-10

**Authors:** Patrick D. Ross, Louise Polson, Marie-Hélène Grosbras

**Affiliations:** Institute of Neuroscience and Psychology, University of Glasgow, Glasgow, United Kingdom; University of Muenster, Germany

## Abstract

To date, research on the development of emotion recognition has been dominated by studies on facial expression interpretation; very little is known about children's ability to recognize affective meaning from body movements. In the present study, we acquired simultaneous video and motion capture recordings of two actors portraying four basic emotions (Happiness Sadness, Fear and Anger). One hundred and seven primary and secondary school children (aged 4–17) and 14 adult volunteers participated in the study. Each participant viewed the full-light and point-light video clips and was asked to make a forced-choice as to which emotion was being portrayed. As a group, children performed worse than adults for both point-light and full-light conditions. Linear regression showed that both age and lighting condition were significant predictors of performance in children. Using piecewise regression, we found that a bilinear model with a steep improvement in performance until 8.5 years of age, followed by a much slower improvement rate through late childhood and adolescence best explained the data. These findings confirm that, like for facial expression, adolescents' recognition of basic emotions from body language is not fully mature and seems to follow a non-linear development. This is in line with observations of non-linear developmental trajectories for different aspects of human stimuli processing (voices and faces), perhaps suggesting a shift from one perceptual or cognitive strategy to another during adolescence. These results have important implications to understanding the maturation of social cognition.

## Introduction

Emotions play an important role in human development. They are an integral part of how our experiences are interpreted, organised and communicated. As such, the identification and recognition of other people's expressed emotions are important skills in the social and emotional development of an individual.

By the age of five, children are able to identify and name all basic emotions [1]; however, emotion recognition is not fully mature until early teenage years [2]–[5]. Existing research also points towards differences across emotions; while happy expressions seem to be interpreted accurately early on, anger and sadness are not recognized with maximum accuracy until late childhood/beginning of adolescence. Likewise, brain imaging studies indicate that the brain networks involved in recognizing others' emotions are not adult-like until late childhood or early adolescence [6]–[8]. Both kinds of studies, behavioural and brain imaging, also suggest non-linearity, with steady increase in performance or specialized brain activity during childhood until the beginning of adolescence followed by no or very little change in emotion recognition during the mid-teenage years and then subsequent improvement from late teenage years until adulthood. Although still ill-understood such non-linearity has been conceptually linked to changes in strategies as well as to structural brain development [8]. Indeed if both changes in social environments (such as starting high-school) and changes in brain maturation of social brain regions occurs in a non-continuous fashion, this might be related to non-linear trends in developmental trajectory of basic social skills.

Most of this research, however, has been conducted when looking at recognition of facial expressions or recognition of speech prosody. There is surprisingly little developmental research with regards to recognising emotion from the body. One could argue that, from an evolutionary point of view, this type of emotion recognition is even more important than those mentioned above. It must be helpful for an individual to be able to predict what somebody else is about to do from their body language, rather than relying on facial expressions [9], [10]. Often, people are seen at a distance, and one can perceive patterns of body motion, posture and gait before cues from facial expression are available. To address the ability of individuals to recognize emotions from body movement and form, several studies have used videos of actors or dancers portraying ‘basic’ emotions of happiness, sadness, fear, disgust and anger [11]–[14]. They all find that adults can identify basic emotions well above chance; although this can be influenced by context [15]. Very few studies have looked at developmental changes in this ability. Two studies investigating the recognition of emotional meaning from dance movements have reported that by 8 years of age children have achieved adult performance in a matching [12] or in a forced-choice task [11]. Using similar stimuli but asking participants to freely name the emotion, Van Meel and colleagues observed that 8-years old performed significantly worse than 12 years old [16]. It could be argued, however, that by using dance, the actions are exaggerated and are a more symbolic representation of the emotion in question. How this finding generalizes to everyday-life body movements remains uncertain.

Furthermore, this research raises questions regarding which body cues allow emotions to be perceived: biological motion direction and speed, or static form [17]. This can be addressed by using ‘point-light displays’ in which the body is represented by a small number of illuminated dots, positioned in such a way as to highlight the motion of the main body parts. When static, the display appears as a meaningless configuration of points; yet, when moving, the display gives a striking impression of a moving body [18]. Point-light displays are sufficient for individuals to identify socially relevant features, such as gender of the actor [19], [20] or, more importantly in regards to this study, affective state [21]–[24]. This may imply that people are able to perceive emotions from kinematic patterns without having to compute the detailed shape of the human form first.

The remarkable efficiency in extracting complex information from animate motion presented in point-light displays suggests that the detection of biological motion has a simple developmental trajectory [25]. Indeed, the ability to extract form from motion develops very early on, with an advantage for biological motion [26]: Infants as young as 12 months can follow the ‘gaze’ of a point-light actor suggesting that they are able to interpret the action depicted [27]; By five years of age, children perform as well as adults for identifying a body from moving dots [28]. However, adding noise to the displays reveals that the sensitivity to biological motion continues to develop during adolescence [29], [30]. Therefore, despite children possessing the ability to extract human form from the point-light displays, we might expect that the more complex analysis necessary to discriminate emotion from fine spatio-temporal patterns will continue to develop into late childhood and adolescence. Adults too recognize emotions better from full-light than from point-light displays, which indicates a benefit from having the full complexity, yet one might expect children to be further impaired from impoverished information in the point-light display. Indeed, during development, social interactions change and inferences from body cues are made from further distances as a child gets older and one could expect that motion information becomes more important. Also, younger children spend a considerable amount of time interacting with the hands of their parents; and hands seem particularly important to convey emotion [27]. Since this information is absent from point-light displays one might also expect children to be further disadvantaged compared to adolescents or adults. In consequence, we were interested in establishing the developmental trajectory of emotion perception from body movements, when only the simplified biological motion is present and additionally study whether the benefit from the full information is similar across ages.

We therefore probed basic emotion recognition from either point-light or full-light displays of short body movements in a sample of 107 primary and secondary school children (4–17 years old). We used angry, fearful, sad and happy expressions as these are the emotions that are most commonly used in emotion research [31]. Importantly, we created both sets of stimuli from the same capture, thus avoiding confound between display condition and variability in emotion portrayal. In addition, contrary to previous studies, which have constrained stimuli into codified emotion expressions used in dance or mime [12], our actors were given more freedom and instructed to execute movements commonly produced when experiencing basic emotions (e.g. shaking fists in anger, retreating in fear, jumping for joy etc. See also [14]).

In summary, we expect that the ability to recognise emotion from bodies will improve during adolescence, possibly in a non-linear trajectory as it has been proposed for the perception of emotion from faces [2]–[4], [11]. Furthermore, the use of both full-light and point-light displays allows us to compare emotion recognition when both form and motion cues are present with performance when only biological motion information is present. Based on previous research with adult participants [14], [21], we expect, on average, poorer recognition when viewing point-light displays compared to full-light displays. Any interaction between age and display condition, however, would indicate that the differential use of socially-relevant visual cues changes during development.

## Methods

### 1.1 Ethics Statement

The study was in line was the Declaration of Helsinki and was approved by the local Ethics Committee (reference: FIMS 00673).

### 1.2. Participants

One hundred and seven primary and secondary school children (aged 4–17 years; *M = *9.89 years; *SD = *3.96, 57 girls) from after-school clubs in the West End of Glasgow (n = 50) and the Shetland Islands (n = 57) participated in the study. Permission was obtained from the managers of the after-school clubs, as well as consent from the children's parents or guardians a week prior to testing from the respective clubs. These clubs offered a homogeneous sample in terms of socio-economic status (assessed with range of tax-bands, level of education and employment figures from Glasgow Census Report Pupils Scotland 2008, and Shetland in Statistics published by Shetland Islands Council Economic Development Unit). All participants understood that participation was voluntary and gave their assent. As a comparison group, we tested a sample of 14 adult volunteers (aged 21–51 years; *M = *32.9 years; *SD = *11.1, 7 women) from the University of Glasgow.

### 1.3. Stimuli

The stimuli were created using a Vicon motion capture system (Vicon Systems, 2009) to capture the movements of two actors. Twelve Vicon MX Series Cameras were connected to two Vicon Ultranet units for the motion capture process.

Two male actors portrayed four emotions (happiness, sadness, fear, and anger). They were wearing dark tight-fitting suits with 31 reflecting balls attached at the location of major joints. They were instructed to move as they would if they were suddenly feeling anger, happiness, sadness or fear respectively, without exaggerating. The actors were given a few practice trials and then we recorded ten captures per actor per emotion. Thus, a total of 80 captures, each lasting between six and ten seconds, were obtained. Each clip was then examined in post processing and seven clips were selected for each emotion, based on the quality of the motion capture (Typically during motion capture, some information will be lost and in some cases reconstruction of the point-light image will be impossible; we selected the clips where reconstruction was possible for all the capture frames). Those 28 clips were subsequently rendered as point-light displays with 16 joint-centred coordinates using the Vicon Nexus software in combination with MATLAB scripts (MATLAB Version 7.12.0, The MathWorks Inc., Natick, MA, 2010). The clips were then cut down to the three seconds containing the main part of the movement using Adobe Premiere Pro.

**Figure 1:**
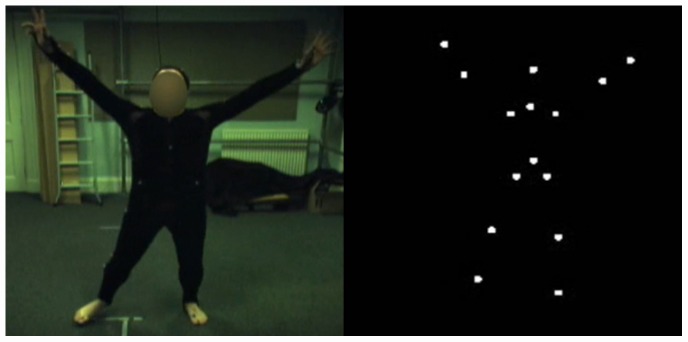
Full-light and Point-light Display Stimuli. Two corresponding frames of a ‘Happy’ clip presented in both Full-light and Point-light display.

The capture clips, which were rendered as point-light displays, were also captured simultaneously as full-light displays using a Basler Digital Video camera. When rendering the point-light displays, the view from which the participant saw the display was set to exactly the same angle and distance from which the full-light video camera was capturing the actors. In other words, both point-light and full-light displays were viewed from exactly the same position (See Figure 1 for screen captures of stimuli). In addition, both sets of videos ran at the same rate of 29 frames/second.

The 28 full-light captures which corresponded to the 28 chosen point-light captures were themselves post-processed using Adobe Premiere Pro. Firstly, the clips were edited to the exact same length (3-seconds long) as their point-light counterparts. Secondly, the actors' faces were blurred out using Gaussian noise so that the participants could not determine any emotion from their facial expressions.

### 1.4. Procedure

Participants sat in front of a 17-inch laptop screen at a distance of about 50 cm. The screen resolution was 1440×900 pixels, with video dimensions of 102×680. E-prime 2.0 software (Psychology Software Tools, Pittsburgh, PA) was used to present the stimuli. Point-light and full-light stimuli were presented in separate blocks, with the order counterbalanced across participants.

Each trial started with a fixation cross displayed for 1 second, followed by one of the videos and then a response screen prompting the participants to decide how the person in the video felt, with a choice of ‘happy’, ‘sad’, ‘scared’ or ‘angry’ respectively. Participants had to indicate their response by pressing a key (c, v, b, or n). Along with the words ‘happy’, ‘sad’, ‘scared’ and ‘angry’, emoticons were used to aid the younger children's responses. Stickers with those emoticons were also placed on the keyboard. Two different associations between keyboard key and respective emotion were used across participants in a counterbalanced order. The prompt for a response stayed on screen until the participant had responded, at which point the next video would start. Each block comprised 20 trials, randomly selected from the 28 clips produced (five per emotion). Participants completed five practice trials, with experimenter's feedback, before starting the experiment. Thus, our independent variables were lighting condition (Full-light and Point-light) as within-subject, and age as a continuous between-subjects variable. Emotion recognition score was the dependent variable.

### 1.5. Analysis

The averaged percentage of correct responses across emotion for each participant was computed. These scores were then entered into separate regression analyses for children and adults to investigate the effects of lighting conditions and age. Changes with age were also assessed using linear regression models. Finally, we analysed recognition performance separately for each emotion. Analyses were conducted using PASW Statistics 18, Release Version 18.0.0 (SPSS, Inc., 2009, Chicago, IL) and Matlab (Mathwork Inc).

## Results

### 2.1 Gender and sample differences in recognition accuracy

We first checked for any gender effect in the group of children across lighting conditions. We found neither a main effect of gender (F(1,105) = .879, *p* = .351) nor an interaction with lighting condition (F(1,105) = .065, *p* = .799). We thus did not consider this factor in subsequent analyses.

We also tested for any differences between the Glasgow and Shetland samples. We found no significant differences between groups (F(1,104) = 2.74, *p* = .101), nor interaction between group and lighting effect (F(1,104) = 0.221, *p = *.64). We thus did not consider this factor in subsequent analyses.

### 2.2. Emotion recognition changes with age and comparison to adults

In adults, as expected, performance under the full-light condition was significantly better than under the point-light condition (t(1,13) = 4.98, *p*<.001) with no effect of age (F(1,13) = 0.06, *p*>.8 for full-light and F(1,13) = 1.17, *p*>.29 for point-light-light). Average performance was 94.3% for full-light and 81.1% for point-light. In comparison, the group of older teenagers (16-17 years old, N = 15) achieved 90% correct and 68.7% correct for full-light and point-light conditions respectively. This was still significantly different from adults (F(1,27) = 12.67, *p*<.001), with no interaction between the age-groups and lighting condition (F(2,27) = 2.32, *p* = 0.14).

**Figure 2:**
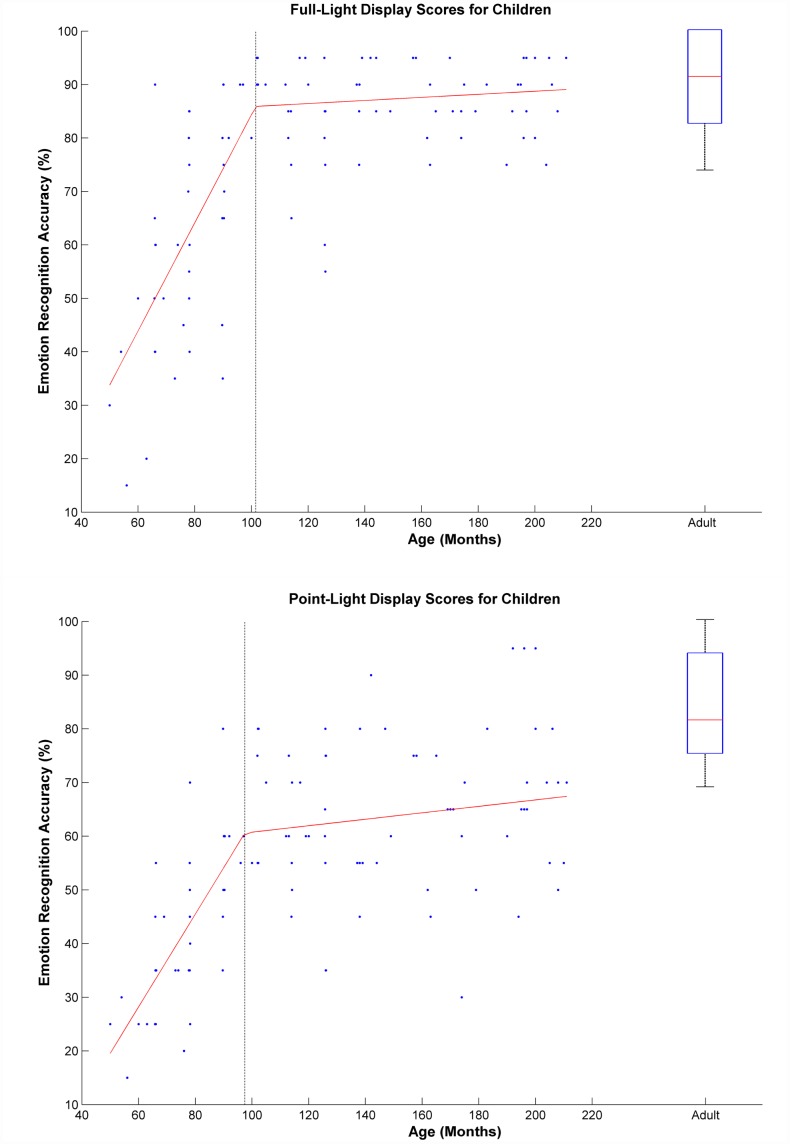
Emotion recognition scores (% correct) for the children recognizing emotion from full-light (top chart) or from point-light (bottom chart) displays of body movements. Children's individual scores are presented as blue dots. The corresponding bilinear trend-lines are presented as a red line, with a black dashed line indicating the position of the knot in both cases. Adults' data are presented as a boxplot in both graphs, representing the median and upper and lower quartiles.

In the children, using a General Linear Model approach, with recognition scores as the dependent variables, and lighting condition and age as fixed and continuous predictor variables respectively, we found a significant effect of age (F(1,105) = 67.32, *p*<.001, partial ŋ^2^ = .391) and lighting condition (F(1,105) = 22.39, *p*<.001, ŋ^2^ = .176). This indicated an increase in performance with age, with children performing significantly better under full-light than point-light (see Figure 2). We found no significant interaction between lighting condition and age (F(1,105) = 1.38, *p* = .24). This indicates that the slopes representing the increase in performance with age were not different in the full-light and point-light conditions.

On inspection of the data, it is clear that it would be better described by two connected linear segments rather than a simple linear model. We therefore used a least-squares approximation algorithm with free-knot splines to determine the position of the knot (i.e. point of change of slope). The results of this piecewise regression analysis are illustrated in Figure 2.

Our data was best explained with two linear segments and a knot at 102 months (RSS = 126.96, R^2^ = .60) for the full-light condition, and a knot at 98 months (RSS = 132.06, R^2^ = .48) for the point-light displays. This model indicates a steep rise in performance (slope β = 1.03 F(1,44) = 42.8, *p*<10^−4^ for full-light condition and β = 0.87 F(1,44) = 55.05, *p*<10^−4^ for point–light condition) until about 8.5 years, followed by a much shallower slope through late childhood and adolescence (Full-Light: β = 0.07, R^2^ = 0.063 F(1,62) = 3.98, *p* = .050; Point-Light: β = 0.11, R^2^ = 0.066 F(1,62) = 4.24, *p*<.05).

### 2.3. Differences across Emotions

**Figure 3:**
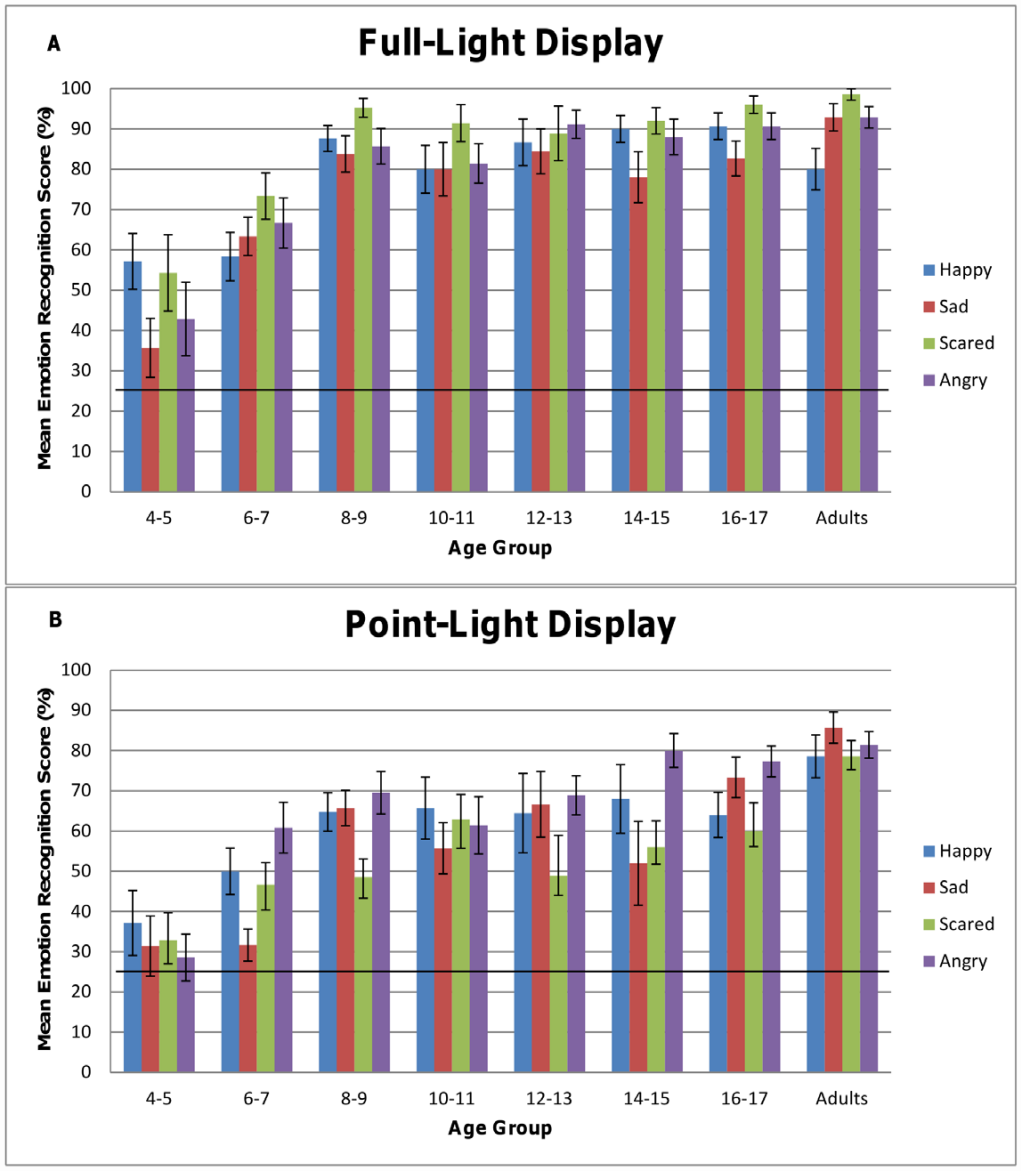
Age and Recognition Score for each Emotion. For display purposes children are grouped by age. Bars representing the mean recognition score (percent correct response) for each group and each emotion are shown under both Full-light Display (A) and Point-light Display (B). Error bars represent SEM. Level of chance marked at 25%. The seven age groups of children comprise 14 (4–5 years old), 24 (6–7 years old), 21 (8–9 years old), 14 (10–11 years old), 9 (12–13 years old), 10 (14–15 years old) and 15 (16–17 years old) subjects respectively.

We also tested differences in recognition scores for the different emotions using a 4×2 mixed design analysis of variance with emotion and lighting condition as within subject factors, age as a continuous between subjects variable and percentage of emotion recognition as dependent variable. We observed no significant difference across emotions and no 3-way or 2-way interactions. The same analysis of the adult's data yielded no significant differences across emotions and neither interaction. For illustrative purposes, we present the mean recognition score for each emotion under both lighting conditions arbitrarily grouping participants by age group (see Figure 3).

## Discussion

Using a forced-choice recognition task, we found that the ability to recognize basic emotions from body movements continues to improve during childhood and adolescence. The data could be well approximated with a bilinear developmental trajectory with a steep increase during childhood followed by a much slower rate of improvement after 8.5 years of age. Similar developmental trajectories were observed when the body movements were shown as full-light displays and when the biological motion was shown through point-lights and no differences were observed across the basic emotions we tested.

### 3.1. Changes in emotion recognition accuracy with age

First our data confirms that children as young as 4 years old perform above chance in decoding affect from body language. Nevertheless, we observed that the refined development of emotion recognition occurs later than what had been described in studies using dance movements as stimuli: both Lagerlof and Djerf [12] and Van Meel et al. [16] suggested that 5-year-old children showed adult-like performance. As dance uses highly codified language to convey emotion, the tasks might have been easier and relied on both true emotion perception and familiarity with such exaggerated movements. Also, the dance stimuli were much longer (between 14 and 96 seconds respectively) than ours, possibly confounded by narrative aspects and thus leading to an easier recognition task. Both of these reasons could account for the fact that we found adult-level performance much later than the aforementioned studies.

Emotion recognition from non-verbal cues has been most often studied using faces or prosody as stimuli. How do our results compare to teenage development for these modalities? While reports have implied that few significant changes in the ability to read facial expressions occur after mid-childhood [32], [5], more and more studies indicate developmental changes well into the teenage years. Using a battery of tests assessing emotion reading from eyes, voice and faces in a cross-sectional design using children aged 9–15, Tonks and colleagues reported that highest performance was achieved at 11 [2]. Using a match-to-sample task with emotional faces or words, McGivern and colleagues showed that reaction times improved until about 13 years of age [33]. A number of older studies using photographs or dynamic faces also points towards adult's level of performance reached between 9 and 12 years of age (rev. in [5]), with differences across studies being probably due to differences in tasks and material [34]. Testing more subtle sensitivity to emotional cues with morphing techniques indicates a protracted development with adolescents aged 14–18 still displaying lower sensitivity to facial expression cues than adults, at least for negative emotions [35]. This suggests that some processes involved in facial expression decoding still develops in late adolescence, which is line with what we observed with our body movements stimuli. This protracted development might be linked to a combination of both a changing external social environment and continuing brain development during that time. Indeed brain regions involved in social and emotion perception, such as the superior temporal cortex, prefrontal cortex [36] and amygdala [37] undergo developmental structural changes at least until the end of the second decade of life. Functional imaging studies also show different brain activity in adolescents and adults while viewing other people moving with an affective connotation [7], [38]. Event-related potentials (ERPs) studies have also shown that the signature of emotion processing when viewing faces is still different from adults at the age of 14 [6]. Thus, it seems that changes are still occurring at the end of the teenage years in the ability to recognize emotions from non-caricatural social stimuli. Further studies are needed to compare emotion recognition across different modalities and how this relates to brain development.

Interestingly, our data show a steep increase in the rate of improvement, followed by a slower improvement throughout late childhood and adolescence. This is in line with other reports of non-continuous development of social cognition abilities. Mann, Diamond and Carey observed that the ability to recognize unfamiliar voices increased steadily from 6 to 10 years of age, reaching adult levels, but then stalling slightly during the beginning of the teenage years to re-attain adult's level at the age of 14 [39]. A similar change in the rate of development during the same age-range has been reported for recognition of facial identity [40], [41] and for recognizing emotion from faces [33]. Using more complex affect processing tasks, such as cartoon matching tasks, Kolb and colleagues reported a slowdown in developmental trend between 8 and 13 years of age [42]. The origin of this change in performance improvement rate at the beginning of adolescence is unknown and is worthwhile investigating further. Similar non-linearity in development of cognitive abilities has been described and proposed to be linked to puberty [33]. Although the brake in improvement occurs around the same time that one would expect to observe pubertal changes, the fact that it happens at the same age for both sexes in all the studies cited, and that the exact age seems different for body, voice and face processing suggests that it is related to maturational events other than hormonal. The fact that this slowdown in development is observed for different aspects of human processing indicates instead that it might be linked to an individual attainment in social skills. It has been suggested that such a change in the slope of the developmental trajectory at the end of childhood/beginning of adolescence might be linked to a shift from one perceptual or cognitive strategy to a new one. During this transition, when the childhood strategy is abandoned, but when the adult strategy is not fully operational yet, performance doesn’t improve or can even be impaired [40]. This explanation is inline with the observation that developmental slowdowns have been reported in other cognitive domains such as working memory [41] or response inhibition [33]. Interestingly, similar trajectories have been observed in fMRI measures of developmental changes of brain activity in relevant regions during emotion processing (supramarginal gyrus, [8]) or during response inhibition (frontal lobe, [43]). Electroencephalography studies have also evidenced non-linearity in long-range synchrony changes pointing towards a reorganization of functional brain networks in late adolescence [44]. Taken together, these data suggests that, perhaps, at the end of childhood individuals start to use new mechanisms to process social stimuli in relation to general changes in executive control abilities, but still do so in an inefficient manner. This would be compatible with the change in slope that we observe in the linear relationship between age and affective body language interpretation.

### 3.2. Differences in Emotion Recognition Accuracy when viewing Full-light Display or Point-light Display

Our results corroborate the previous findings that basic emotions are identifiable from body movements, even when initial form information is eliminated by using point-light displays. The observation that emotion recognition is more accurate when viewing full-light displays compared to point-light displays replicates the results of both Dittrich et al's and Atkinson et al's studies [21], [14]. Yet, unlike previous studies which used a computerised point-light display model, our results were achieved using stimuli in which body movements portrayed in the full-light and point-light displays were identical, as they were created from the same footage. This eliminated the possible confound of differences in movement between these conditions. Furthermore, as we used a computer model in the creation of the point-light displays, we were able to create an ‘internal skeleton’ of point-lights. That is, that rather than point-light displays being achieved by attaching reflective strips to an actor's clothing, our light points could be artificially placed on the exact positions of the actors' bones and joints. This further minimised any unwanted form information, which may have been included in the point-light displays of other studies.

We found that the developmental trajectory of recognition scores was similar for emotion recognition from both kinds of stimuli, with the difference between the two not changing significantly across ages. This advantage of full-light over impoverished point-light stimuli might be due to over reliance on form information, which doesn’t change significantly during childhood and adolescence. This suggests that emotion processing from form and motion information develop at the same time.

### 3.3. Difference across emotions

Previous studies have shown that children aged 4–5 are able to name and recognize all basic emotions from facial expressions [1] and pointed towards differences across emotions. Happiness is most often reported as the emotion most easily recognised by young children, while sadness recognition seems to develop later [32], [13], [11], (but see [12]). In our sample of primary and secondary school children, we did not observe any specific advantage for happiness. On average, sadness was less accurately recognised than the three other emotions, but this was not statistically significant. These discrepancies regarding the rate of development of other basic emotions might be related to tasks and stimuli factors, as well as inter-individual characteristics. In our experiment for example, solving the forced-choice task could rely on development of executive functions. As our experiment was a forced-choice between the four emotions, the process of elimination could have been used in order for the participants to deduce which emotion was presented, particularly in the case of an ambiguous clip (the head in our point-light display clips was represented by only one light point, meaning that some motion information, shaking of the head etc., was lost in a few of the sadness and angry clips). In a review paper on the development of deductive reasoning, Jansson states that logical and deductive reasoning is a characteristic of the concrete operational period, that is, between 7 and 11 years old [45]. This could influence how children solved the four alternatives forced-choice task, and perhaps mask differences across emotions.

## Conclusions

This is the first study to our knowledge that explores the development of the ability to recognize emotion from naturalistic body cues presented as full-light and point-light displays during childhood and adolescence. Our data indicates that, like adults, children recognize emotion better from full-light displays than from point-light displays. This could be due to an over reliance on body form information in emotion recognition from an early age, which impacts upon social processing strategies. Furthermore, a change in these strategies could explain the change in rate of improvement: after a steep increase until 8.5 years of age, the ability to recognize body language then improves at a much slower rate through late childhood and adolescence. We believe that this research has important implications for understanding emotional development in children, both from typical and atypical populations.
